# The synergetic effect of selenium or zinc oxide nanoparticles with chromium on mitigating thermal stress for sustainable production and improving antioxidant capacity and inflammatory cytokines of growing rabbits

**DOI:** 10.5194/aab-68-43-2025

**Published:** 2025-01-06

**Authors:** Ibrahim T. El-Ratel, Khaled H. El-Kholy, Soma M. Elgmmal, Sara Fikry Fouda, Abdel-Khalek E. Abdel-Khalek, Mahmoud A. Hassan, Mahmoud M. Azzam, Mahmoud Alagawany, Antonia Lestingi

**Affiliations:** 1 Department of Animal, Poultry, and Fish Production, Faculty of Agriculture, Damietta University, 34517 Damietta, Egypt; 2 Department of Poultry Production, Faculty of Agriculture, Mansoura University, 35516 Mansoura, Egypt; 3 Department of Animal Production, Faculty of Agriculture, Mansoura University, 35516 Mansoura, Egypt; 4 Agricultural Research Center, Animal Production Research Institute, Dokki, 12619 Giza, Egypt; 5 Department of Animal Production, College of Food and Agricultural Sciences, King Saud University, Riyadh, Saudi Arabia; 6 Poultry Department, Faculty of Agriculture, Zagazig University, 44511 Zagazig, Egypt; 7 Department of Veterinary Medicine, University of Bari Aldo Moro, S.P. per Casamassima km 3, 70010, Valenzano, BA, Italy

## Abstract

This study was conducted to evaluate the impacts of selenium nanoparticles (SeNPs), zinc oxide nanoparticles (ZnONPs), and a combination of SeNPs and chromium (Cr) or ZnONPs and Cr on growth, caecal microbiota, antioxidant capacity in blood and liver tissue, and inflammatory cytokines in heat-stressed rabbits. A total of 100 newly weaned APRI rabbits were randomly divided into five homogeneous groups. A basal diet containing no feed additives (0 g per kg diet) was given to the first group, and the second, third, fourth, and fifth groups were given a diet supplemented with 0.3 mg SeNPs, 20 mg ZnONPs, 0.3 mg SeNPs and 1.5 mg Cr, and 20 mg ZnONPs and 1.5 mg Cr per kg diet, respectively. At 10 and 14 weeks of age, the live body weight (LBW) of rabbits was higher ( P < 0.05) in all treatments, while LBW at 10 weeks of age was higher ( P < 0.05) in combination groups. All treatments increased daily body weight gain in the age intervals of 6–10 and 6–14 weeks ( P<0.05 ). Daily body weight gain was increased ( P < 0.05) in combination groups at the age interval of 6–10 weeks. Feed intake was only increased for rabbits in the ZnONP–Cr group at age intervals of 10–14 weeks. The feed conversion ratio was significantly improved in all treatments at 6–10 and 6–14 weeks of age compared to the control. Haemoglobin was increased ( P<0.05 ) in diets supplemented with ZnONPs and SeNP–Cr or ZnONP–Cr combinations. The platelet count was only increased (P < 0.05) by the ZnONP–Cr combination compared to other groups. Serum total proteins, total antioxidant capacity, superoxide dismutase, glutathione peroxidase, IgA, IgM, nitric oxide, and lysozyme were increased, while serum total cholesterol and triglycerides, alanine transaminase, malondialdehyde, myeloperoxidase, tumour necrosis factor (TNF- α ), and interleukin 4 (IL-4) were reduced by all treatments. The total antioxidant capacity in liver tissue was higher, and malondialdehyde was lower in all treatment groups. Albumin was significantly increased, while glucose, creatinine, and urea were significantly decreased in response to ZnONPs and SeNP–Cr or ZnONP–Cr combinations compared with the other groups. Dietary addition of SeNPs–Cr or ZnONPs–Cr significantly reduced interferon-gamma (IFN- γ ) concentration. The caecal activity was increased, while the *Escherichia coli* (*E. coli*) count decreased considerably in all treatments compared to the control. In conclusion, SeNPs or ZnONPs with chromium as trace elements of growing rabbits can be recommended as an effective intervention to mitigate the negative impacts of heat stress (HS) by enhancing growth performance, promoting metabolic processes, and boosting immunity.

## 1 Introduction

As a non-ruminant herbivore, rabbits are characterized by high-fibre diets and are considered hindgut digesters. Rabbits are important in Egypt's meat production (Amer et al., 2019). Compared with other livestock, rabbits have a rapid reproductive cycle and growth rate, high meat quality, and high genetic selection potential (Kumar et al., 2018; El-Ratel et al., 2023). Rabbits are susceptible to heat stress (HS) and heat anxiety in hot climates (Madkour et al., 2020). Under HS conditions, changes in oxidative stress, acid–base imbalance, and immunosuppression lead to a reduction in growth performance, including weight gain, feed consumption, feed utilization, and viability of rabbits (Amer et al., 2019; Abdel-Wareth et al., 2022). Also, HS causes oxidative DNA, protein, and lipid damage, leading to an increase in the reactive oxygen species (ROS) generation and a decrease in the body's antioxidant defence system's ability to remove toxins (Prasad and Bao, 2019).

To eliminate the harmful impacts of HS, different feeding strategies by dietary supplementation with phytogenics, amino acids, enzymes, trace minerals, vitamins, prebiotics, or probiotics have been used to enhance animal growth performance (Alagawany et al., 2021a; Abdelnour et al., 2022; El-Ratel et al., 2020). Relating to this, minerals have an important role as nutraceuticals in animal production for their requirement to cover the optimal response of animals to physiological and metabolic actions by increasing the enzymatic and hormonal activities and regulating the acid–base balance and osmotic homeostasis of animals (Weyh et al., 2022; El-Gindy et al., 2023; Mohamed et al., 2023).

Chromium (Cr) is a micronutritional element for metabolizing carbohydrates, lipids, proteins, nucleic acids, and physiological processes (El-Kholy et al., 2017; Mohamed et al., 2023). Cr is a component of chromodulin and an oligopeptide low-molecular-weight Cr-binding substance, which plays a central role in the mechanism of insulin signalling auto-amplification (Amer et al., 2019), leading to increased glucose transport and decreased HS via a reduced level of stress proteins (Piray and Foroutanifar, 2021).

Zinc (Zn) is one of the most crucial and nutritional trace minerals and is included in nucleic acid biosynthesis and cellular division processes (Alagawany et al., 2021b). Also, it has a vital role in physiological functions in the body and has an ability as an antioxidant by scavenging the production of free radicals (Martemucci et al., 2022). It can be used as a dietary supplement to promote bone development, growth, enzyme structure, and immunity in poultry (Madkour et al., 2022; Abdel-Wareth et al., 2022). Moreover, it is pivotal in the metabolism of proteins, fats, and carbohydrates (Kechrid and Bouzerna, 2004) and in gut health, consequently increasing feed utilization (Surai et al., 2017).

Selenium (Se), as an essential trace element, is vital for animal rearing in HS conditions. Se is important for body metabolism, with enzymatic antioxidant properties such as glutathione peroxidase (GPx) to prevent lipid peroxides and ROS from causing cellular damage (Amer et al., 2019). Dietary Se in an appropriate amount is crucial for physiological and biological functions, including productive and reproductive efficiency, immune response, antioxidative action, stress protection, and metabolism of hormones in animals (Qazi et al., 2019; Abdelnour et al., 2022; Liang et al., 2022; El-Ratel et al., 2023). Organic Se has lower toxicity and higher efficacy than inorganic Se in animals under HS conditions (Kim and Kil, 2020).

Nanotechnology is applied in feeding animals, and the most basic approaches in this area are nanoformulations with 1–100 nm dimensions. Nanoformulations have increased bioavailability, specific surface area, surface centre activity, catalytic and adsorptive capacity, and low toxicity (El-Ratel et al., 2023). They are supplemented with diets at low levels more efficiently than their native forms (Hassan et al., 2017), so nanoformulations can be easily assimilated into the digestive tract (Konkol and Ojnarowski, 2018).

Dietary supplementation with a combination of Zn, Se, and Cr was reported to enhance broiler growth performance (Ghasemi et al., 2020). Addition of Zn–Cr in diets up to 3160 mg kg^−1^ can increase productive efficiency and the physiological status of chicks (Ognik et al., 2020; Zaghari et al., 2023). A dietary combination of Zn–Cr and organic Se reduced the impaired effects of HS on broilers via increased metabolic processes and improved growth efficiency (Mohamed et al., 2023).

In the current study, we hypothesized that nanoparticles of trace elements alone or a combination can mitigate the negative impacts of HS conditions on productive performance parameters, health status markers, and immune response of growing rabbits. Therefore, this study aimed to investigate the modulatory effects of the dietary inclusion of Se or Zn in nanoparticle forms, or a combination of Se nanoparticles and Cr (native form) or Zn nanoparticles and Cr (native form), on the growth performance, caecal activity, haemato-biochemical parameters, antioxidant capacity in blood and liver tissue, immunoglobulins, inflammatory cytokines, and carcass traits of growing rabbits reared under HS conditions.

## 2 Materials and methods

The Animal, Poultry, and Fish Production Department, Faculty of Agriculture, Damietta University, Egypt, supervised the experimental work for this study at a commercial rabbit farm in Mansoura, Dakahlia Governorate. The European Community norms (EU Directive 2010/63/EU) for the scientific care and use of animals guided all operations.

### 2.1 Animals

In this work, the experimental animals included 100 APRI rabbits (Egyptian line, Red Baladi × V-line species, selected for litter weight according to Abou Khadiga et al., 2010). They were weaned at 42 d of age and had an average body weight of 694.72±3.324 g. All weaned rabbits underwent a 7 d adaptation period before the start of the trial.

The animals were housed individually in galvanized wire battery cages ( 50×45×40 cm) with feeders and automatic drinking nipples. During the experiment, rabbit cages were placed in an open facility with natural ventilation (windows and ceiling fans) and a photoperiod of 16 h of light and 8 h of darkness. All rabbits were subjected to similar management, hygiene, and environmental settings throughout the experiment.

### 2.2 Experimental design

The rabbits were randomly separated into five groups. A basal diet containing no feed additives (0 g per kg diet) was given to the first group, and the second, third, fourth, and fifth groups were given a diet supplemented with 0.3 mg selenium nanoparticles (SeNPs), 20 mg zinc oxide nanoparticles (ZnONPs), 0.3 mg SeNPs and 1.5 mg Cr (SeNPs–Cr), and 20 mg ZnONPs and 1.5 mg Cr (ZnONPs–Cr) per kg diet, respectively. The experiment lasted 8 weeks during July and August. The basal diet was a full pelleted meal free of antibiotics, commercially used to feed growing rabbits. The ingredients and chemical analysis of the basal diet are reported in Table 1.

**Table 1:** Ingredients and chemical analysis of the basal diet fed to the growing rabbits.

Ingredient	%
Berseem hay	30.05
Barley grain	24.60
Wheat brain	21.50
Soybean meal	17.50
Molasses	3.00
Dicalcium phosphate	1.60
Limestone	0.95
DL-Methionine	0.15
Sodium chloride	0.30
Vitamins & mineral premix^*^	0.35
Total	100
Analysed composition (%, on dry matter (DM) basis)	
Organic matter	91.42
Crude protein	17.36
Crude fibre	12.37
Ether extract	2.23
Nitrogen-free extract	59.46
Ash	8.58

^*^ Each 1 kg contains vitamin A (15 000 IU), vitamin E (100 mg), vitamin B1 (10 mg), vitamin K3 (21 mg), vitamin B2 (40 mg), vitamin B6 (15 mg), vitamin B12 (0.1 mg), pantothenic acid (100 mg), niacin (200 mg), biotin (0.5 mg), folic acid (10 mg), and choline chloride (5000 mg). Each 1 kg contains manganese (800 mg), zinc (600 mg), iron (300 mg), copper (40 mg), iodine (500 mg), selenium (100 mg), and cobalt (100 mg).

### 2.3 Climate conditions

The ambient temperature (AT) and relative humidity (RH) were measured daily at 14:00 PM. An automatic thermohygrometer was used (OF 14:140, H 10 %–99 %; TFA Dostmann GmbH & Co. KG, Wertheim, Germany). During the experiment, temperature–humidity index (THI) values were determined using the formula by Marai et al. (2022): THI = Tdb - [(0.31 - 0.31(RH)] × [(Tdb - 14.4)], where Tdb is the dry bulb temperature (°C). THI readings range from < 27.8 (lack of HS) to >30.0 (extremely severe HS). The moderate HS is 27.8–28.9.

### 2.4 Growth performance parameters

During the trial, live body weight (LBW) was measured at 6 (initial), 10, and 14 weeks (final) of age, and the average daily weight growth was determined at age intervals of 6–10, 10–14, and 6–14 weeks. Furthermore, feed intake was recorded separately per day, and the average feed intake over the same intervals was calculated by weighing the residuals of daily given feed. The feed conversion ratio (FCR; g feed / g gain) was also computed for the same age intervals.

### 2.5 Blood samples

At the end of the trial (14 weeks old), blood samples were carefully collected from five rabbits in each group. After topical anesthesia with 4 % xylocaine, samples were obtained from the marginal ear vein and placed in two test tubes, one with heparin (as an anticoagulant) and the other without.

Whole blood samples were collected in heparinized tubes and used to assess some haematological parameters such as haemoglobin (Hb), the count of red (RBCs) and white (WBCs) blood cells, platelet count, and packed cell volume (PCV) using a blood haematology analyser. The non-heparinized tubes were kept at room temperature for 2 h to clot. Blood samples were centrifuged (T32c; Janetzki, Wallhausen, Germany) at 700× g for 15 min to isolate blood serum in 1.5 mL Eppendorf tubes. The serum was then refrigerated at -20 °C for further biochemical analysis.

Colorimetric analysis was used to assess the concentrations of total protein (TP), albumin (AL), glucose, total cholesterol (TC), triglycerides (TGs), urea, and creatinine in the serum. Commercial chemical kits (BioSystems S.A., Barcelona, Spain) were used for this procedure. However, the globulin (GL) concentration was computed by subtracting the AL values from the corresponding TP values. The activities of aspartate (AST) and alanine (ALT) transaminase in blood serum were determined using commercial kits (BioSystems S.A., Barcelona, Spain). Serum redox status, immunoglobulins, and inflammatory cytokine indicators were determined following the manufacturer directives.

Blood serum samples were tested for total antioxidant capacity (TAC), glutathione (GSH), superoxide dismutase (SOD), glutathione peroxidase (GPx), and malondialdehyde (MDA) using commercial kits (Diagnostic, Egypt). In addition, serum myeloperoxidase activity was measured using commercial kits (MBS724170, MyBioSource, San Diego, CA, USA).

ELISA kits measured immunoglobulin concentrations in blood serum, specifically immunoglobulins (IgG, IgM, and IgA). Blood serum levels of interferon-gamma (IFN- γ ) (MBS2601171), tumour necrosis factor (TNF- α (MBS7612133), and interleukin 4 (IL-4) (MBS733925) were measured using commercial sandwich ELISA kits (MyBioSource, San Diego, USA). Nitric oxide (NO) and lysosome activity were also measured in blood serum.

#### 2.5.1 Total antioxidant capacity and malondialdehyde levels in liver tissue

After scarification, liver tissue samples were collected from five rabbits per group, homogenized with 10 % w/v potassium phosphate buffer (pH 7.4) solution, and centrifuged at 700× g for 20 min. TAC and MDA levels in supernatants were measured using commercial chemical kits (Diagnostic and Research Reagents, Dokki, Giza, Egypt) and a spectrophotometer (Shimadzu, Japan).

### 2.6 Carcass characteristics

Five rabbits were randomly chosen from each group after the growth period had ended (14 weeks old). Rabbits were fasted for 12 h and weighed before killing by a trained person. After slaughter and thorough bleeding, the viscera, tail, and pelt were removed, followed by the carcass, head, liver, heart, lung, spleen, and kidneys, which were weighed and calculated as a percentage of pre-slaughter weight. The dressing percentage was obtained by dividing the net carcass weight (hot-dressed) by the pre-slaughter weight times 100.

### 2.7 Caecal characteristics

After slaughter, caecal contents samples were collected and filtered using an OP-110. The concentration of ammonium nitrogen (NH_3_-N) and volatile fatty acids (VFAs) was measured. The total microbial and *Escherichia coli* (*E. coli*) counts in the caecal content were measured. The pH of the caecal contents was tested using a Radelkis pH meter (Hungary).

### 2.8 Statistical analysis

The Levene and Shapiro–Wilk tests were used to assess normality and variance homogeneity. The data were statistically evaluated using one-way ANOVA (PROC ANOVA; SAS, 2012 version 8, Cary, NC, USA) to determine the influence of treatment on the various parameters studied. Duncan's multiple range test was used to compare means with a significance threshold of P < 0.05. The results were presented as means ± standard error of the mean (SEM).

## 3 Results

### 3.1 Climate conditions

During the experiment, the rabbits experienced heat stress, averaging 31.62±0.24 °C, 65.10 % RH ± 0.85 % RH, and 29.76±0.20 THI (Table 2).

**Table 2:** The mean values of ambient temperature, relative humidity, and temperature–humidity index during the experimental period.

Item	July	August	Overall
AT (°C)	32.14±0.19	31.11±0.25	31.62±0.24
RH (%)	63.83±1.11	66.38±2.18	65.10±0.85
THI value	30.15±0.13	29.36±0.19	29.76±0.20

RH: relative humidity. AT: ambient temperature. THI: temperature–humidity index.

### 3.2 Growth performance

Table 3 shows the effects of dietary supplementation with SeNPs, ZnONPs, and a combination of SeNPs–Cr or ZnONPs–Cr on the growth performance of heat-stressed rabbits. At 10 and 14 weeks of age, rabbits had greater LBW ( P<0.05 ) in all treatments compared to the control group. At 10 weeks of age, the combination group had higher LBW ( P < 0.05) than the SeNP or ZnONP groups. However, the variations in LBW across treatment groups were not significant. All treatments significantly increased average daily gain ( P < 0.05) at 6–10 and 6–14 weeks of age compared to the control group. At 6–10 weeks of age, combination groups showed a better average daily increase ( P<0.05 ) compared to SeNP or ZnONP groups. However, the treatment groups did not affect the average daily increase between 10 and 14 weeks of age. At the age interval of 10–14 weeks, only rabbits in the ZnONP–Cr group increased their feed consumption compared to the control group, although this did not differ substantially from the other treatment groups. However, there was no significant effect of treatment on rabbit feed consumption at 6–10 and 6–14 weeks. The feed conversion ratio improved considerably in all treatments compared to the control group at 6–10 and 6–14 weeks of age but not at 10–14 weeks.

**Table 3:** Effects of dietary different sources of trace mineral supplementation on growth performance and feed utilization of growing rabbits exposed to heat stress conditions.

Variables	Control	Treatment (mg per kg diet)				P value
				SeNP–chromium	ZnONP–chromium	
		SeNPs	ZnONPs	combination	combination	
Live body weight (g) at						
6 weeks of age	692.64 ± 3.543	694.40 ± 2.946	693.36 ± 4.822	694.68 ± 3.608	698.52 ± 2.959	0.8148
10 weeks of age	1382.12 ± 6.004^c^	1468.92 ± 4.107^b^	1476.12 ± 6.009^b^	1485.20 ± 4.622^a^	1487.60 ± 3.719^a^	< 0.0001
14 weeks of age	2007.96 ± 6.375^b^	2103.44 ± 5.249^a^	2107.52 ± 23.718^a^	2124.92 ± 7.609^a^	2123.80 ± 5.697^a^	<0.0001
Average daily gain (g)						
6–10 weeks of age	24.62 ± 0.146^c^	27.66 ± 0.112^b^	27.96 ± 0.103^b^	28.23 ± 0.089^a^	28.18 ± 0.076^a^	< 0.0001
10–14 weeks of age	22.35 ± 0.131	22.66 ± 0.150	22.549 ± 0.865	22.85 ± 0.206	22.72 ± 0.189	0.9364
Overall (6–14 weeks of age)	23.49 ± 0.092^b^	25.16 ± 0.082^a^	25.252 ± 0.453^a^	25.54 ± 0.111^a^	25.45 ± 0.090^a^	< 0.0001
Feed intake (g d^−1^) at						
6–10 weeks of age	68.40 ± 1.034	67.84 ± 1.212	69.72 ± 1.389	68.72 ± 0.939	68.12 ± 0.876	0.7860
10–14 weeks of age	110.80 ± 1.656^b^	112.92 ± 1.146^a,b^	113.88 ± 1.836^a,b^	113.88 ± 1.871^a,b^	115.72 ± 1.008^a^	0.2584
Overall (6–14 weeks of age)	89.88 ± 1.374	90.24 ± 10648	89.44 ± 1.915	87.84 ± 1.368	90.88 ± 1.417	0.7077
Feed conversion ratio (g feed / g gain)						
6–10 weeks of age	2.78 ± 0.04^a^	2.45 ± 0.042^b^	2.494 ± 0.050^b^	2.43 ± 0.0342^b^	2.417 ± 0.293^b^	< 0.0001
10–14 weeks of age	4.96 ± 0.076	4.98 ± 0.064	6.65 ± 1.789	4.99 ± 0.099	5.10 ± 0.073	0.4993
Overall (6–14 weeks of age)	3.83 ± 0.059^a^	3.58 ± 0.065^b^	3.58 ± 0.117^b^	3.44 ± 0.052^b^	3.57 ± 0.057^b^	0.0084

^a,b,c^ Means ( n=20 ) not sharing a common superscript in a row are significantly different ( P<0.05 ). SeNPs, selenium nanoparticles (0.3 mg per kg diet); ZnONPs, zinc nanoparticles (20 mg kg^−1^); SeNP–chromium combination (0.3 mg SeNPs per kg diet + 1.5 mg chromium per kg diet); ZnONP–chromium combination (20 mg ZnONPs per kg diet + 1.5 mg chromium per kg diet).

**Table 4:** Effects of dietary different sources of trace mineral supplementation on carcass traits of growing rabbits exposed to heat stress conditions.

Variables	Control	Treatment (mg per kg diet)				P value
				SeNP–chromium	ZnONP–chromium	
		SeNPs	ZnONPs	combination	combination	
Pre-slaughter LBW (g)	1997 ± 24.72	2105 ± 14.460	2126 ± 8.375	2124 ± 14.171	2154 ± 7.429	0.0956
Dressing (%)	54.26 ± 0.914^b^	55.38 ± 0.546^a,b^	55.79 ± 8.274^a,b^	55.61 ± 0.647^a,b^	57.29 ± 0.485^a^	0.0542
Head	4.98 ± 0.145	5.07 ± 0.128	5.21 ± 0.132	5.19 ± 0.164	5.16 ± 0.148	0.8856
Liver	3.11 ± 0.236	3.21 ± 0.124	3.34 ± 0.138	3.490 ± 0.114	3.62 ± 0.064	0.1447
Heart	0.30 ± 0.041	0.33 ± 0.032	0.31 ± 0.028	0.28 ± 0.020	0.33 ± 0.046	0.8856
Kidney	0.63 ± 0.046	0.63 ± 0.060	0.61 ± 0.044	0.64 ± 0.028	0.61 ± 0.021	0.9756
Lung	0.65 ± 0.052	0.63 ± 0.045	0.59 ± 0.070	0.62 ± 0.051	0.63 ± 0.054	0.9717
Spleen	0.07 ± 0.020	0.09 ± 0.015	0.11 ± 0.012	0.09 ± 0.027	0.10 ± 0.024	0.6856

^a,b^ Means not sharing a common superscript in a row are significantly different ( P<0.05 ). SeNPs, selenium nanoparticles (0.3 mg per kg diet); ZnONPs, zinc nanoparticles (20 mg kg^−1^), SeNP–chromium combination (0.3 mg SeNPs per kg diet + 1.5 mg chromium per kg diet), ZnONP–chromium combination (20 mg ZnONPs per kg diet + 1.5 mg chromium per kg diet).

### 3.3 Carcass criteria

The effect of treatments on carcass features, such as percentage weight of head, liver, heart, kidney, lung, and spleen, was not significant (Table 4). The dressing rate was higher ( P < 0.05) in rabbits fed a diet enriched with the ZnONP–Cr combination compared to the control group, but it did not differ substantially from other treatment groups.

### 3.4 Haemo-biochemical parameters

Table 4 shows how different sources of trace minerals affect haematological and biochemical markers. Diets supplemented with ZnONPs and combined with SeNPs–Cr or ZnONPs–Cr resulted in considerably higher Hb concentrations ( P < 0.05). Platelet count increased considerably ( P < 0.05) with the ZnONP–Cr combination compared to the SeNP and control groups. Treatments had no significant effect on RBC and WBC counts, as well as the PCV (%). In terms of blood biochemistry, adding SeNPs, ZnONPs, and SeNP–Cr or ZnONP–Cr combinations to growing rabbit diets significantly increased TP concentrations, while it decreased total cholesterol and triglyceride concentrations and ALT activity in serum compared to the control group. In response to ZnONPs and SeNP–Cr or ZnONP–Cr combinations, albumin concentrations increased, while glucose, creatinine, and urea concentrations decreased ( P < 0.05) compared to the other groups. Dietary supplements did not significantly affect globulin, the AL/GL ratio, or AST activity.

**Table 5:** Effect of dietary different sources of trace mineral supplementation on haemato-biochemical parameters of growing rabbits exposed to heat stress conditions.

Variables	Control	Treatment (mg per kg diet)				P value
				SeNP–chromium	ZnONP–chromium	
		SeNPs	ZnONPs	combination	combination	
Hematological parameters						
Hb (g dL^−1^)	10.66 ± 0.36^c^	10.97 ± 0.15^b,c^	11.67 ± 0.175^a,b^	11.63 ± 0.42^a,b^	11.87 ± 0.161^a^	0.0291
RBCs (10^6^ mm^−3^)	5.10 ± 0.199	± 5.06 0.080	5.19 ± 0.061	5.27 ± 0.102	5.22 ± 0.099	0.7455
WBCs (10^3^ mm^−3^)	6.57 ± 0.174	6.04 ± 0.064	6.18 ± 0.039	6.12 ± 0.067	6.16 ± 0.309	0.1940
Platelets (10^3^ mm^−3^)	270.31 ± 8.3^b^	269.69 ± 12.5^b^	290.52 ± 3.511^a,b^	294.19 ± 8.2^a,b^	301.32 ± 3.9^a^	0.0343
PCV (%)	31.82 ± 1.233	29.10 ± 1.816	31.22 ± 1.469	30.64 ± 1.002	31.03 ± 1.938	0.7750
Biochemical parameters						
Total proteins (TP, g dL^−1^)	6.29 ± 0.078^b^	6.83 ± 0.090^a^	6.88 ± 0.058^a^	6.87 ± 0.035^a^	6.95 ± 0.081^a^	< 0.0001
Albumin (AL, g dL^−1^)	3.65 ± 0.103^c^	3.86 ± 0.047^c^	3.89 ± 0.122^a,b^	4.06 ± 0.083^a,b^	4.16 ± 0.075^a^	0.0077
Globulin (GL, g dL^−1^)	2.63 ± 0.173	2.97 ± 0.090	2.99 ± 0.126	2.80 ± 0.086	2.79 ± 0.133	0.2894
AL/GL ratio	1.42 ± 0.131	1.31 ± 0.047	1.32 ± 0.093	1.46 ± 0.076	1.51 ± 0.093	0.4619
Glucose (mg dL^−1^)	96.29 ± 3.993^a^	90.09 ± 1.440^a^	81.17 ± 2.191^b^	80.80 ± 2.369^b^	77.17 ± 1.535^b^	0.0001
Triglycerides (mg dL^−1^)	191.42 ± 4.928^a^	170.15 ± 6.402^b^	154.10 ± 6.181^b,c^	151.21 ± 7.033^c^	149.32 ± 4.940^c^	0.0003
Total cholesterol (mg dL^−1^)	80.32 ± 1.843^a^	63.20 ± 2.377^b^	61.11 ± 2.015^b^	58.97 ± 2.739^b^	57.48 ± 2.495^b^	< 0.0001
Creatinine (mg dL^−1^)	1.00 ± 0.035^a^	0.90 ± 0.038^a,b^	0.85 ± 0.029^b,c^	0.82 ± 0.035^b,c^	0.77 ± 0.035^c^	0.0017
Urea (mg dL^−1^)	40.94 ± 1.757^a^	40.07 ± 1.900^a^	35.17 ± 1.304^b^	35.02 ± 0.923^b^	31.02 ± 1.645^b^	0.0011
AST (IU)	31.25 ± 1.687	29.81 ± 2.503	26.11 ± 1.510	26.05 ± 1.648	25.16 ± 1.036	0.0875
ALT (IU)	54.34 ± 1.956^a^	45.52 ± 1.608^b^	45.69 ± 2.353^b^	43.91 ± 1.695^b^	42.27 ± 3.578^b^	0.0158

^a,b,c^ Means ( n=5 ) not sharing a common superscript in a row are significantly different ( P < 0.05). AST, aspartate transaminase; ALT, alanine transaminase. SeNPs, selenium nanoparticles (0.3 mg per kg diet); ZnONPs, zinc nanoparticles (20 mg kg^−1^), SeNP–chromium combination (0.3 mg SeNPs per kg diet + 1.5 mg chromium per kg diet), ZnONP–chromium combination (20 mg ZnONPs per kg diet + 1.5 mg chromium per kg diet).

**Table 6:** Effect of dietary different sources of trace mineral supplementation on antioxidant capacity in blood and liver tissue of growing rabbits exposed to heat stress conditions.

Variables	Control	Treatment (mg per kg diet)				P value
				SeNP–chromium	ZnONP–chromium	
		SeNPs	ZnONPs	combination	combination	
Antioxidant capacity in blood serum						
TAC (ng mL^−1^)	0.33 ± 0.021^b^	0.44 ± 0.024^a^	0.48 ± 0.031^a^	0.52 ± 0.039^a^	0.53 ± 0.031^a^	0.0011
GSH (mg dL^−1^)	12.86 ± 0.977	13.0 ± 0.681	13.65 ± 0.207	13.69 ± 0.249	14.13 ± 0.230	0.5017
SOD (U mL^−1^)	0.258 ± 0.019^*c*^	0.33 ± 0.024^b^	0.34 ± 0.017^b^	0.38 ± 0.017^a,b^	0.43 ± 0.033^a^	0.0006
GSH-Px (mg dL^−1^)	4.82 ± 0.071^b^	5.00 ± 0.091^a^	5.15 ± 0.051^a^	5.34 ± 0.060^a^	5.25 ± 0.039^a^	< 0.0001
MDA (nmol mL^−1^)	11.43 ± 0.362^a^	9.61 ± 0.356^b^	8.65 ± 0.266^b,c^	8.48 ± 0.504^c^	8.24 ± 0.256^c^	< 0.0001
Myeloperoxidase (ng mg^−1^ protein)	4.61 ± 0.179^a^	4.21 ± 0.115^b^	3.95 ± 2.104^b^	3.99 ± 0.059^b^	3.94 ± 0.042^b^	0.0005
Antioxidant capacity in liver tissue						
TAC liver (nmol mL^−1^)	0.916 ± 0.034^b^	1.14 ± 0.033^a^	1.16 ± 0.024^a^	1.18 ± 0.046^a^	1.29 ± 0.155^a^	0.0311
MDA liver (nmol mL^−1^)	4.16 ± 0.085^a^	3.75 ± 0.068^b^	3.59 ± 0.112^b^	3.51 ± 0.148^b^	3.46 ± 0.067^b^	0.0003

^a,b,c^ Means ( n=5 ) not sharing a common superscript in a row are significantly different ( P < 0.05). SeNPs, selenium nanoparticles (0.3 mg per kg diet); ZnONPs, zinc nanoparticles (20 mg kg^−1^), SeNP–chromium combination (0.3 mg SeNPs per kg diet + 1.5 mg chromium per kg diet), ZnONP–chromium combination (20 mg ZnONPs per kg diet + 1.5 mg chromium per kg diet). TAC, total antioxidant capacity; GSH, glutathione; SOD, superoxide dismutase; GSH-Px, glutathione peroxidase; MDA, malondialdehyde.

### 3.5 Antioxidant capacity in blood serum and liver tissue

The results in Table 6 reveal that nutritional treatment substantially affects the redox state in the serum and liver tissue of developing rabbits under heat stress. When compared to the control group, all treatments caused raised serum TAC, SOD, and GPx activity and decreased serum MDA and myeloperoxidase activity. TAC levels were significantly higher, while MDA levels were significantly lower in the liver tissue of all treatment groups compared to the control.

### 3.6 Immunity and inflammatory cytokines

Table 7 shows how different sources of trace minerals affect immunoglobulins and inflammatory cytokines. Dietary supplementation with all supplements significantly raises IgA and IgM concentrations compared to the control ( P<0.05 ). However, supplementation had no significant effect on IgA concentrations. Dietary supplements significantly ( P<0.05 ) enhanced nitric oxide and lysozyme while decreasing TNF- α and IL-4 levels compared to the control group. However, only the dietary addition of SeNP–Cr or ZnONP–Cr combinations significantly lowered IFN- γ concentration relative to the control group but did not differ substantially from the SeNP and ZnONP groups.

**Table 7:** Effect of dietary different sources of trace mineral supplementation on immunoglobulins and inflammatory cytokines of growing rabbits exposed to heat stress conditions.

Variables	Control	Treatment (mg per kg diet)				P value
				SeNP–chromium	ZnONP–chromium	
		SeNPs	ZnONPs	combination	combination	
Immunoglobulins						
IgG (ng mL^−1^)	43.87 ± 2.066	51.20 ± 1.020	53.62 ± 0.907	53.66 ± 1.467	53.92 ± 1.274	0.1082
IgA (ng mL^−1^)	17.89 ± 1.600^b^	21.92 ± 2.022^a^	22.27 ± 1.838^a^	23.34 ± 1.622^a^	23.11 ± 1.230^a^	< 0.0001
IgM (mg dL^−1^)	66.94 ± 2.697^b^	80.39 ± 2.150^a^	82.70 ± 1.716^a^	83.92 ± 2.015^a^	83.36 ± 1.846^a^	< 0.0001
Inflammatory cytokines						
Nitric oxide ( µ mol L^−1^)	48.81 ± 1.890^c^	55.14 ± 1.286^b^	59.76 ± 2.100^a,b^	61.54 ± 2.101^a^	61.77 ± 2.285^a^	0.0006
TNF- α (pg mL^−1^)	83.27 ± 2.176^a^	71.68 ± 4.392^b^	67.66 ± 0.112^b^	66.29 ± 2.273^b^	66.21 ± 1.711^b^	0.0017
IL-4 (pg mL^−1^)	107.89 ± 2.432^a^	97.79 ± 2.330^b^	94.04 ± 2.100^b^	93.71 ± 1.182^b^	90.87 ± 2.882^b^	0.0011
IFN- γ (pg mL^−1^)	72.23 ± 6.286^a^	65.25 ± 1.558^a,b^	62.89 ± 2.830^a,b^	61.68 ± 1.911^b^	58.71 ± 1.204^b^	0.0133
Lysozyme (pg mL^−1^)	1.91 ± 0.025^c^	2.31 ± 0.035^b^	2.49 ± 3.252^a^	2.52 ± 0.026^a^	2.54 ± 0.027^a^	< 0.0001

^a,b,c^ Means ( n=5 ) not sharing a common superscript in a row are significantly different ( P < 0.05). SeNPs, selenium nanoparticles (0.3 mg per kg diet); ZnONPs, zinc nanoparticles (20 mg kg^−1^), SeNP–chromium combination (0.3 mg SeNPs per kg diet + 1.5 mg chromium per kg diet), ZnONP–chromium combination (20 mg ZnONPs per kg diet + 1.5 mg chromium per kg diet). IgG, immunoglobulin G; IgM: immunoglobulin M; TNF- α : tumour necrosis factor; IL-4: interleukin 4; IFN- γ , interferon-gamma.

### 3.7 Caecal characteristics

Table 8 shows that the amounts of ammonia-N and VFAs in the caecal contents of growing rabbits were increased by supplementation of feed additives. At the same time, the *E. coli* count was reduced in all treatments compared to the control group. Feed additives did not significantly affect the caecal length or pH value of caecal contents.

**Table 8:** Effect of dietary different sources of trace mineral supplementation on caecal activity and *E. coli* count of growing rabbits exposed to heat stress conditions.

Variables	Control	Treatment (mg per kg diet)				P value
				SeNPs–chromium	ZnONPs–chromium	
		SeNPs	ZnONPs	combination	combination	
Cecum length (cm)	11.65 ± 0.596	11.51 ± 0.701	12.19 ± 0.495	12.47 ± 0.501	12.17 ± 0.431	0.7160
Ammonia nitrogen (NH3-N)	14.87 ± 0.569^b^	16.71 ± 1.229^a^	18.66 ± 0.045^a^	18.77 ± 1.318^a^	19.05 ± 0.840^a^	0.0507
Volatile fatty acids (VFAs)	8.05 ± 0.345^b^	8.94 ± 0.160^a^	9.09 ± 0.1474^a^	9.25 ± 0.087 ^a^	9.23 ± 0.126^a^	0.0016
Caecum pH value	5.79 ± 0.100	5.73 ± 0.257	5.77 ± 0.108	5.75 ± 0.088	5.70 ± 0.194	0.9951
*Escherichia coli* (*E. coli*)	8.46 ± 0.598^a^	7.51 ± 0.401^b^	7.52 ± 0.607^b^	7.37 ± 0.347^b^	7.35 ± 0.307^b^	0.0060

^a,b^ Means ( n=5 ) not sharing a common superscript in a row are significantly different ( P<0.05 ). SeNPs, selenium nanoparticles (0.3 mg per kg diet); ZnONPs, zinc nanoparticles (20 mg kg^−1^), SeNP–chromium combination (0.3 mg SeNPs per kg diet + 1.5 mg chromium per kg diet), ZnONP–chromium combination (20 mg ZnONPs per kg diet + 1.5 mg chromium per kg diet).

## 4 Discussion

Rabbits are vulnerable to HS and have few sweat glands, making it difficult to cool their bodies (Marai et al., 2002). Hot climates impact rabbit productivity and physiological states (Jaen-Tellez et al., 2021). Our study found that growing rabbits experienced HS during the experimental period, as shown by the computed THI value of 29.76±0.20 . Dietary trace mineral supplementation is required to ameliorate the negative effects of HS on poultry (Abdel-Wareth et al., 2022; El-Ratel et al., 2023; Mohamed et al., 2023). In this study, the hypothesis is that supplementation with different trace minerals, such as SeNPs, ZnONPs, SeNPs–Cr, or ZnONPs–Cr, may have a positive influence on the growth performance, feed utilization, and health status of growing rabbits, hence mitigating the harmful effects of HS. These trace minerals are excellent feed additions with various potential medicinal effects, including antioxidant, antibacterial, antiviral, anti-inflammatory, and hepatoprotective qualities for rabbits growing under HS conditions (Abdel-Wareth et al., 2022; El-Ratel et al., 2023; Mohamed et al., 2023). In the current study, dietary supplementation of SeNPs, ZnONPs, and SeNPs–Cr or ZnONPs–Cr combinations improved rabbit growth performance parameters at 6–10 weeks of age as compared to the control group, in terms of higher LBW at 10 weeks of age, higher daily gain, and no significant changes in feed intake at the age interval of 6–10 weeks. Increased LBW of rabbits at 10 weeks of age, minor changes in daily gain at 10–14 weeks, and increased feed intake in treatment groups resulted in no significant differences in feed conversion ratio between treatment groups and the control group under HS circumstances. However, adding the ZnONP–Cr combination increased the dressing rate ( P < 0.05) in rabbits compared to the control group but did not differ substantially from other treatment groups. In this regard, Mohamed et al. (2023) suggest that trace elements like Zn–Cr and organic Se may significantly reduce the effects of HS on broilers by improving growth performance and carcass features. In addition, Jain et al. (2020) found that Zn–Cr and Se increased the feed conversion ratio in broilers. In growing rabbits, dietary supplementation with ZnONPs can reduce the deleterious effects of HS on productivity (Kamel et al., 2020; Abdel-Wareth et al., 2022). Dietary ZnONPs have been demonstrated to help reduce the deleterious effects of HS in poultry (Ramiah et al., 2019; Shokraneh et al., 2020). Many authors have demonstrated the promising favourable benefits of ZnONPs for animal performance (Abdelnour et al., 2021; Cui et al., 2021). Se nanoparticles, particularly when manufactured biologically, increased the growth performance and carcass features of growing rabbits during thermal stress (Sheiha et al., 2020), and feeding Se nanoparticles improves rabbit productivity during HS (El-Badry et al., 2019). Dietary supplementation with SeNPs may improve the growth performance of broilers raised under HS conditions (El-Deep et al., 2016). The improvement in weight gain may be attributed to the fact that trace minerals can increase intestinal trace element absorption by reducing interference from substances that form insoluble complexes with ionic trace elements, thereby improving their bioavailability and body weight development (Jain et al., 2020). Furthermore, dietary trace element supplementation may increase feed utilization and nutrient absorption compared to the control diet in broilers with poor growth performance under HS, which spent more energy to adapt to the stressor than to grow (Alwaleed et al., 2021).

Enhanced growth performance in this study may be connected to improved feed consumption and nutrient digestibility. In this regard, trace element supplementation increases nutritional digestibility, resulting in a higher FCR (Sahin et al., 2009). In the current study, SeNPs, ZnONPs, and a combination of SeNPs–Cr or ZnONPs–Cr improved growth performance due to the trace elements' critical role in the molecular structure of enzymes and proteins, serving as co-enzymes and activators that are primarily involved in physiological functions (Nguyen et al., 2021). Zn is widely recognized for increasing the activity of the insulin-like growth factor and growth hormone genes, as well as for controlling hunger and improving nutrient performance and digestibility (Ibrahim et al., 2017). Cr also plays a role in the metabolism of glucose, lipids, proteins, and nucleic acids, in addition to its growth-promoting actions, and it enhances the biological function of insulin-sensitive cell receptors by boosting their binding activity (Mohamed et al., 2023).

In this study, heat-stressed rabbit diets fortified with ZnONPs and a combination of SeNPs–Cr or ZnONPs–Cr increased Hb concentration and platelet count significantly ( P < 0.05) compared to other groups. This study improved heat-stressed broiler chicks' haematological parameters by dietary addition of Se yeast alone or in combination with Zn–Cr (Mohamed et al., 2023). The significant variations in Se, Zn, or Cr related to haematological parameters could be attributed to Se's positive influence on increasing Hb concentration (Mohamed et al., 2023) and Zn's participation in erythropoiesis, which leads to an increase in Hb when broilers are fed zinc-supplemented diets. Zn is also a catalytic agent for α -aminolevulinic acid dehydrogenase, an enzyme implicated in heme formation (Aksu et al., 2010). The increase in Hb content in treatment groups could be attributed to Zn–Cr and Se–Cr's antioxidant capabilities and their ability to enhance enzyme synthesis, stability, and activity in the body (Mohamed et al., 2023). In terms of blood biochemicals, adding SeNPs, ZnONPs, and SeNP–Cr or ZnONP–Cr combinations to growing rabbit diets significantly increased total protein concentration, decreased total cholesterol and triglyceride concentrations, and decreased serum ALT activity when compared to the control group. ZnONPs and SeNP–Cr or ZnONP–Cr combinations significantly raised albumin concentrations, while they decreased glucose, creatinine, and urea concentrations ( P<0.05 ) compared to other groups. Reducing ALT activity implies improved liver function, mitigating the negative effects of a heated environment (Kew, 2002; Sheiha et al., 2020). These improvements in rabbit serum biochemistry may be related to zinc being a component of Zn metalloenzymes, which maintain protein structural integrity (Chrastinová et al., 2015). Se can reduce the formation of thiobarbituric-acid-reactive substances while increasing the activity of glutathione S-transferase and total sulfhydryl groups, which are necessary components of the enzyme glutathione peroxidase, which in turn prevents lipid and protein oxidation (El-Demerdash, 2004). This impact could be attributable to all supplements reducing cholesterol absorption and synthesis in the poultry gut (Abd El-Hack et al., 2019). Se nanoparticles can improve the lipid profile, possibly due to its protective properties against HS.

Furthermore, selenium-coated nanostructures are helpful in the treatment of fatty liver disease by increasing oral bioavailability and improving the hypoglycemic effects of phytogenesis (Hosnedlova et al., 2018). Chromium is an essential mineral that is an integral component of chromodulin and is required for insulin activity (Vincent, 2000). The Cr can enhance glucose transporter for transfer from the cytoplasm to the cell membrane by activating insulin receptors and facilitating glucose entrance into cells (Vincent 2015). Furthermore, Cr is a key component of the glucose tolerance factor, which influences glucose metabolism, lipids, proteins, and nucleic acids by enhancing insulin activity (Hayirli, 2005). Combining Zn–Cr and organic Se increased broiler chicks' blood biochemical and metabolic responses, including high total protein and LDH (lactate dehydrogenase) and low glucose, triglycerides, LDL (low-density lipoprotein), creatinine, and uric acid (Mohamed et al., 2023). ZnONP supplementation effectively ( P < 0.05) reduced blood cholesterol, creatinine, and urea concentrations, as well as AST and ALT activity, in developing rabbits under HS circumstances (Abdel-Wareth et al., 2022). Furthermore, SeNPs play an important role in enhancing rabbit blood metabolites under HS circumstances (Bashar et al., 2022) and renal function by lowering uric acid and creatinine concentrations (Sheiha et al., 2020). In line with our findings, Cr-propionate supplementation reduced serum hyperglycemia in broiler chicks compared to the control group but did not affect AST and ALT activity (Arif et al., 2019).

In rabbits exposed to HS, serum antioxidant enzyme activity was lowered, and MDA levels increased, resulting in organ damage and decreased productivity (El-Ratel et al., 2020, 2023). In the current study, all treatments raised serum TAC content, SOD, and GPx activities while decreasing serum MDA levels and myeloperoxidase activity as compared to the control group. TAC levels were significantly greater in liver tissue, but MDA levels were significantly lower in all treatment groups compared to the control. HS is widely known to induce ROS formation, linked to apoptosis and other disorders caused by lipid peroxidation damage to DNA, proteins, and cell phospholipid membranes (Abdel-Wareth et al., 2022). Trace elements may reduce the negative effects of HS on animal health by reducing free radicals and inhibiting lipid peroxidation (Ai et al., 2009; El-Ratel et al., 2023). Supplementation with Zn–Cr and/or organic Se significantly increased serum antioxidant activity by boosting TAC content and decreasing MDA levels compared to the non-supplemented group of broiler chicks (Mohamed et al., 2023). Also, supplementing broilers' diets with organic forms of 0.3 mg Se, 2 mg Cr, and 40 mg Zn kg^−1^ (individually or in combination) significantly increased their antioxidant responses to SOD and MDA during the hot summer season (Rao et al., 2016).

Increasing the TAC content and activity of SOD and GPx, as well as the reduction in MDA levels in serum of supplemented rabbits under HS circumstances, demonstrated significant benefits of SeNP–Cr or ZnONP–Cr combinations as dietary supplements in limiting the detrimental effects of HS. Zn is a powerful indirect antioxidant that prevents free radical formation and slows oxidative processes (Vlaicu et al., 2022). Furthermore, Zn supplementation reduces free radicals since it is a component of SOD and GSH, which play an important role in the formation of metallothionein and act as free radical scavengers (Lee, 2018; Liang et al., 2022). Accordingly, Zn is a key element that must be included in poultry diets under HS (Liang et al., 2022). These findings suggested that Zn is an anti-heat stress agent and is primarily found in the forms of Zn monocarbonate, Zn sulfate monohydrate, Zn pyridine acid, bacitracin Zn, granular coated bacitracin Zn, and amino acid Zn (Liang et al., 2022).

On the other hand, dietary Se nanoparticle supplementation improved antioxidant activities in rabbits by enhancing GSH activity and decreasing MDA levels compared to the heat-stressed group (Sheiha et al., 2020). Se is a crucial component of various Se proteins, including GPx and thioredoxin reductase (Zhou et al., 2013); hence, the improved antioxidant capability may be attributable to inducible Se-dependent antioxidant enzymes. In this regard, the GPx is a prominent phase II detoxification enzyme that may reduce the degree of lipid peroxidation, and dietary inclusion of a sufficient quantity of Se was discovered to be critical in scavenging oxidation and boosting immunity in rabbits (Liang et al., 2022). Similarly, trace minerals offer potential medicinal activity against oxidation, bacteria, viruses, and inflammation, in addition to hepatoprotective qualities (Abdel-Wareth et al., 2023; El-Ratel et al., 2023; Mohamed et al., 2023).

Under HS circumstances, a significant negative effect on the immunological response in rabbits was observed via the hypothalamic–pituitary–adrenal axis (Elazab et al., 2022) to elicit glucocorticoid functions as an anti-immunity element (Liang et al., 2022). Increasing glucocorticoid concentrations changes both the cellular and humoral immune systems. As a result, HS has a negative impact on rabbit production, including decreased immunity and susceptibility to infections (Marai et al., 2002). In our investigation, dietary supplementation with SeNPs, ZnONPs, and a combination of SeNPs–Cr or ZnONPs–Cr boosted the cellular and humoral immune systems of heat-stressed developing rabbits, as evidenced by a significant rise in serum IgA, IgM, and nitric oxide concentrations, as well as lysozyme activity. The observed increase in lysozyme activity could be attributed to its enzymatic degenerative ability to remove infections (Hashem et al., 2021).

Furthermore, the beneficial effects of these trace minerals on immunity can be related to their diverse biological capabilities, which include antioxidant, antibacterial, antiviral, anti-inflammatory, and hepatoprotective qualities (Abdel-Wareth et al., 2022; El-Ratel et al., 2023; Mohamed et al., 2023). Along with their favourable effects on the immune response of growing rabbits, trace minerals can decrease the de novo production of inflammatory cytokines and, hence, inflammation reactions. HS increases the apical content of pro-inflammatory cytokines.

Abd El-Hack et al. (2020) found that IFN- γ and IL-4 increased intestinal permeability to infections. Our study found that dietary SeNPs, ZnONPs, and SeNPs–Cr or ZnONPs–Cr combinations significantly reduced pro-inflammatory cytokines TNF- α and IL-4 in heat-stressed developing rabbits. The dietary addition of SeNP–Cr or ZnONP–Cr combinations dramatically lowered IFN- γ levels relative to the control group but did not differ significantly from the SeNP and ZnONP groups. These findings suggested that these minerals had potential anti-inflammatory properties, indicating their efficacy in improving the health of animals suffering from HS. SeNPs (25 or 50 mg per kg diet) improved inflammatory cytokines in developing rabbits exposed to temperature stress (Sheiha et al., 2020). Improving animal tolerance to environmental stressors is an important step toward eradicating or avoiding viral illnesses by encouraging immune enhancers and stimulants (Alagawany et al., 2017).

As a result, we investigated the influence of various supplements on the caecal activity of growing rabbits and discovered that ammonia-N and VFA concentrations in caecal contents increased significantly, with a drop in *E. coli* count by all dietary supplements compared to the control diet. The antimicrobial action of trace elements in decreasing *E. coli* count in caecal contents may be related to the presence of bioactive substances that hinder microbial growth and disrupt specific metabolic processes. These findings showed that employing SeNPs, ZnONPs, or a combination of SeNPs–Cr and ZnONPs–Cr as feed additives was safe for microbial fermentation in rabbit cecum.

## 5 Conclusions

Overall, the results of this study revealed that SeNPs, ZnONPs, and the combination of SeNPs–Cr or ZnONPs–Cr in heat-stressed young rabbits may increase growth rate, carcass characteristics, haematological parameters, biochemical indicators, antioxidant levels, immunology, and inflammatory cytokines. This could be owing to the enhanced bioavailability of certain trace elements after being fabricated in nanoform. SeNP–Cr or ZnONP–Cr combinations are active components that play essential roles as thermoregulatory agents and regulate several physiological activities in rabbits during their growth period. More research is needed to determine how these trace elements affect the reproductive performance of male and female rabbits.

## Data Availability

The data presented in this study are available on request from the corresponding author.
